# *Lactobacillus murinus* Mediates Multi-Target Protection to Alleviate Cyclophosphamide-Induced Intestinal Injury and Immune Suppression Through the Gut–Metabolism–Immune Axis

**DOI:** 10.3390/biom16070957

**Published:** 2026-06-29

**Authors:** Jingna Wu, Nan Pan, Xiaoting Chen, Lexuan Qi, Hui Huang, Xiaoya Qu, Zhiyu Liu

**Affiliations:** 1 Xiamen Key Laboratory of Marine Medicinal Natural Products Resources, Fujian Universities and Colleges Engineering Research Center of Marine Biopharmaceutical Resources, Xiamen Medical College, Xiamen 361023, China; wjn@xmmc.edu.cn (J.W.); qilexuan28@gmail.com (L.Q.); 202511710071@jmu.edu.cn (H.H.); quxiaoya@mail.ustc.edu.cn (X.Q.); 2 Fisheries Research Institute of Fujian, Xiamen 361013, China; npan01@qub.ac.uk (N.P.); xtchen@jmu.edu.cn (X.C.)

**Keywords:** *Lactobacillus murinus*, cyclophosphamide, intestinal barrier, short-chain fatty acids, metabolomics, immune regulation

## Abstract

The protective effects of *Lactobacillus murinus* on chemotherapy-related intestinal injury and immune imbalances were explored by establishing a cyclophosphamide (CTX)-induced mouse model of immunosuppression. CTX treatment led to intestinal barrier destruction, exacerbated local inflammation, and significantly reduced short-chain fatty acid levels (especially butyrate), accompanied by systemic immune suppression. *Lactobacillus murinus* intervention, especially at medium and high doses, dose-dependently repaired the intestinal barrier, inhibited inflammatory responses, restored levels of metabolites such as butyrate, and systematically regulated splenic immune cell proportions, restoring the CD4^+^/CD8^+^ balance. Metabolomic analysis further revealed that, at different doses, this regulation affected distinct metabolic pathways: low doses enhanced glutathione and purine metabolism, medium doses restored folate and steroid hormone metabolism, and high doses promoted fatty acid β-oxidation and galactose metabolism, forming a multi-level metabolic protective network. This suggests that *L. murinus* can alleviate chemotherapy-induced intestinal mucositis and mitigate systemic immune suppression through a dual local anti-inflammatory and systemic immune-regulatory effect, with potential mechanisms related to butyrate-mediated regulation of the “metabolism–immune axis,” providing evidence for probiotic-assisted chemotherapy.

## 1. Introduction

Cyclophosphamide (CTX), a classic alkylating agent and immunosuppressant, is widely used for the clinical treatment of malignant tumors and autoimmune diseases. However, its severe dose-limiting toxicity—especially its capacity to induce bone marrow suppression and gastrointestinal reactions—substantially limits its efficacy and impairs patient quality of life [1]. Traditionally, CTX toxicity has been primarily attributed to its direct cytotoxic effects on rapidly proliferating cells. However, increasing evidence suggests that the toxicity of chemotherapy drugs occurs via a complex pathological process involving multiple organs and systems, with mechanisms extending beyond direct cytotoxic effects [2].

In recent years, metabolic dysregulation has been regarded as a key feature and driving factor of chemotherapy-induced toxicity [3]. Chemotherapeutic drugs can induce systemic metabolic reprogramming in the host, affecting important pathways such as one-carbon metabolism and fatty acid β-oxidation; this is closely related to tissue damage and immune suppression [4,5]. Meanwhile, as a metabolically active organ highly sensitive to chemotherapy, the intestine exhibits villous atrophy, downregulation of tight junction protein expression, and increased barrier permeability when exposed to chemotherapeutic drugs, thereby exacerbating local and systemic inflammatory responses [6,7]. Notably, barrier disruption is often accompanied by gut microbiota dysbiosis, leading to reduced levels of its metabolites, particularly short-chain fatty acids (SCFAs), represented by butyrate [8,9,10]. As a key signaling molecule linking the gut microenvironment to systemic immune regulation, butyrate exerts anti-inflammatory effects, maintains the epithelial barrier integrity, and regulates T cell differentiation through different pathways, such as by inhibiting histone deacetylases (HDACs) and activating G protein-coupled receptors (GPCRs) [11,12]. This suggests that targeting specific beneficial metabolic pathways or supplementing with key microbial metabolites may be effective strategies for alleviating chemotherapy side effects.

Our research group previously found that polysaccharides from *Bangia fuscopurpurea* significantly enhance *Lactobacillus murinus* abundance in the intestines of immunosuppressed mice. Notably, *L. murinus* exerted bidirectional immunomodulatory effects: it enhanced immune function under immunosuppressive conditions by promoting macrophage phagocytosis and increasing the levels of interleukin-2, tumor necrosis factor-alpha (TNF-α), and interferon-gamma (IFN-γ), while preventing excessive inflammation under inflammatory conditions by stimulating interleukin-10 (IL-10) expression and promoting M2 macrophage polarization. These observations prompted us to directly investigate whether *L. murinus* itself could protect against CTX-induced injury. *Lactobacillus murinus* is a common member of the *Lactobacillus* genus that colonizes the intestines of mammals, particularly in mouse and rat intestines, where it occupies an important ecological niche. These bacteria can aid in maintaining the intestinal microecological balance through mucosal colonization, nutrient competition, and ecological niche occupation [13]. In addition to potential colonization-resistance effects, many *Lactobacillus* strains have been shown to directly or indirectly influence the abundances of host metabolites and exert anti-inflammatory and immune-regulatory effects [14,15]. However, whether *L. murinus* can systematically alleviate CTX-induced multi-organ toxicity and whether its protective effects depend on intestinal barrier repair, the regulation of specific host metabolic pathways, and subsequent restoration of immune homeostasis have not been systematically explored. Furthermore, differences in the effects of *L. murinus* across doses and the associated core mechanisms remain to be elucidated; this is significant for understanding the mechanisms underlying red algal polysaccharide effects and for developing precise chemotherapy-adjunct strategies.

Therefore, we aimed to systematically explore the multi-target protective effects of different doses of *L. murinus* on CTX-treated mice, as well as the associated mechanisms, within the “gut–metabolism–immune axis” theoretical framework. By integrating immunohistochemistry, flow cytometry, tissue and serum cytokine detection, targeted SCFA metabolomics, and untargeted metabolomics, we comprehensively analyzed the regulatory effects of *L. murinus* on intestinal barrier integrity, host systemic metabolic profiles, and local and systemic immune states, revealing the core mechanisms of *L. murinus* in alleviating CTX toxicity. These findings provide a solid mechanistic basis for the application of *L. murinus* as a metabolic-immune regulator for chemotherapy-adjunct therapy and pioneer a new therapeutic strategy for managing chemotherapy-induced toxicity through gut microbiota-based interventions.

## 2. Materials and Methods

### 2.1. Materials

#### 2.1.1. Experimental Animals

Male BALB/c mice of SPF grade (aged 4–6 weeks; weighing 18 ± 2 g) were purchased from Shanghai SLAC Laboratory Animal Co., Ltd. (license number: SCXK 2017-0005, Shanghai, China). The mice were acclimatized in an SPF-grade animal facility for 1 week and maintained under standard controlled conditions: a 12 h light/dark cycle, an ambient temperature of 25 °C, and free access to food and water.

#### 2.1.2. Drugs and Reagents

*Lactobacillus murinus* (BNCC194688) was obtained from BeNa Culture Collection; RIPA lysis buffer was sourced from Beyotime Biotechnology (Shanghai, China); fetal bovine serum was from Hyclone (Logan, UT, USA); RPMI1640 culture medium was from Corning; acrylamide, bis-acrylamide, and APS were from Amresco (Solon, OH, USA); the pre-stained protein marker was from Fermentas (Vilnius, Lithuania); bromophenol blue was from Ruibio (Changzhou, China); the enhanced chemiluminescence (ECL) substrate was from Millipore (Burlington, MA, USA); and protease inhibitors and phosphatase inhibitors were from Roche (Basel, Switzerland). Antibodies for flow cytometry, including anti-CD45-PECY5 (E-AB-F1136G), anti-CD11C-FITC (E-AB-F0991C), anti-CD86-PE (E-AB-F0994D), anti-CD80-APC (E-AB-F0992E), anti-CD3-FITC (E-AB-F1013C), anti-CD4-PECY7 (E-AB-F1097H), anti-CD8-APC (E-AB-F1104E), anti-CD19-PE (E-AB-F0986D), and anti-CD49b-PECY7 (E-AB-F1116H), were all purchased from Wuhan Elabscience Biotechnology Co., Ltd. (Wuhan, China). Anti-TNF-α (rabbit A0277) and anti-IL1β (rabbit A20527) were purchased from Abclonal Biotechnology Co., Ltd. (Wuhan, China); anti-IFN-γ (rabbit R381656) and anti-Mucin2 (rabbit R381746) were purchased from Chengdu Zhengneng Biotechnology Co., Ltd. (Chengdu, China); anti-IL10 (mouse 60269-1-IG), anti-ZO1 (21773-1-AP), anti-Occludin (27260-1-AP), and anti-Claudin1 (28674-1-ap) were purchased from Wuhan Sanying Biotechnology Co., Ltd. (Wuhan, China); and anti-GAPDH (Mouse D190090), HRP-labeled goat anti-mouse IgG (D110087-0100), and HRP-labeled goat anti-rabbit IgG (D110058-0100) were purchased from Shenggong Biotechnology (Shanghai) Co., Ltd. (Shanghai, China). TEMED, Accutase, butanol, acetonitrile, and SCFA standards were purchased from Sigma–Aldrich (Shanghai, China). Ammonium acetate, ammonia solution, methanol, and formic acid were purchased from Aladdin (Shanghai, China).

### 2.2. Methods

#### 2.2.1. Experimental Animal Grouping

For the first animal trial, all mice were allocated to four distinct groups (*n* = 7). The groups were designated as follows: control (CON), low-dose (LD), medium-dose (MD), and high-dose (HD). Mice in the CON group received a daily oral gavage of 200 µL of sterile saline, whereas those in the LD, MD, and HD groups were treated with 200 µL of an *L. murinus suspension* at 1 × 10^5^, 1 × 10^7^, and 1 × 10^9^ CFU/mL (200 µL/dose), respectively, once daily for 30 days. During the administration process, the condition of each mouse, especially their activity and appetite, was observed regularly, and weight changes were recorded every 3 days; 24 h following the final administration, all mice were fasted for 12 h and were subsequently euthanized in compliance with ethical guidelines. Whole blood samples were obtained for comprehensive blood count assessments using a hematology analyzer (Kangyu Medical HF3800, Taian, China), serum was collected for ELISA to detect relevant cytokine levels, and the small intestine tissue was harvested for a histological evaluation via hematoxylin-eosin (H&E) staining.

For the second animal trial, all mice were allocated to five distinct groups (*n* = 7). The groups were designated as follows: control group (CON group), CTX group, and CTX + low-dose *L. murinus* (L group), CTX + medium-dose *L. murinus* (M group), and CTX + high-dose *L. murinus* (H group). The experimental protocol was implemented as follows. During the initial 3-day period, mice in the CON group received daily intraperitoneal injections of saline, while those in the CTX, L, M, and H groups received daily intraperitoneal injections of CTX (80 mg/kg). Following the injection phase, mice in the CON and CTX groups were gavaged with 200 µL of sterile saline via oral gavage each day, whereas mice in the L, M, and H groups received daily oral administrations of 200 µL of an *L. murinus* suspension, with concentrations of 1 × 10^5^, 1 × 10^7^, and 1 × 10^9^ CFU/mL, respectively, for 30 days. Animals were fasted for 12 h and euthanized at the 24-h time point after the last dose. Samples including spleens, serum, small intestine tissue, and intestinal contents were harvested for flow cytometry, ELISA, Western blot/immunohistochemistry, and metabolomics/SCFA detection, respectively.

All animal experiments were conducted in accordance with the animal ethics protocol approved by the Institutional Animal Care and Use Committee of Xiamen Medical College (Approval No. [20230308004]). All procedures were performed in compliance with the ARRIVE 2.0 guidelines and institutional regulations governing animal welfare.

#### 2.2.2. Flow Cytometric Analysis

Spleen tissues from mice were collected, washed several times with PBS, and cut into 1 × 1 mm pieces using ophthalmic surgical scissors. Next, 1 mL of Accutase was added for cell digestion, and during digestion, the tissue was pipetted several times with a Pasteur pipette until no visible tissue remained. After terminating the digestion with 2 mL of complete medium and centrifuging (134× *g*, 5 min), the supernatant was discarded. The cells were then resuspended in 2 mL of PBS, pelleted again under identical centrifugation conditions, and the supernatant was removed.

Approximately 1 × 10^6^ cells were taken for surface staining. First, an Fc receptor blocker (anti-CD16/32) was incubated with the sample at 4 °C for 10 min. Then, a pre-titrated optimized mixture of fluorescent antibodies was added, including the following: anti-CD45/CD3/CD19, anti-CD45/CD3/CD4/CD8, anti-CD45/CD80/CD86/CD11C, and anti-CD3/CD19/CD45/CD49b. Following a 30-min incubation in the dark at 4 °C, the cells were washed by centrifugation at 134× *g* for 5 min and then analyzed by flow cytometry using a BD FACS Celesta.

Total splenocytes were initially gated based on forward scatter height vs. side scatter to exclude debris and cell aggregates. Leukocytes were identified as CD45^+^ cells within the lymphocyte/monocyte scatter gate. T cells were defined as CD45^+^CD3^+^ cells. Among these, CD4^+^ T cells were identified as CD45^+^CD3^+^CD4^+^ cells, whereas CD8^+^ T cells were identified as CD45^+^CD3^+^CD8^+^. B cells were defined as CD45^+^CD19^+^ cells. Natural killer (NK) cells were defined as CD45^+^CD3^−^CD19^−^CD49b^+^ cells. Dendritic cells (DCs) were defined as CD45^+^CD11c^+^CD80^+^CD86^+^ cells.

For T-cell subsets (CD4^+^ and CD8^+^ T cells), percentages were calculated within the CD45^+^CD3^+^ T cell gate. The percentage of B cells was calculated within the CD45^+^CD19^+^ B-cell gate. NK-cell frequency was determined as the proportion of CD49b^+^ cells within the CD45^+^CD3^−^CD19^−^ gate. DC frequency was determined as the proportion of CD86^+^CD80^+^ double-positive cells within the CD45^+^CD11c^+^ gate.

#### 2.2.3. ELISA

Blood was collected via retro-orbital bleeding under isoflurane anesthesia. Whole blood was allowed to clot at room temperature for 2 h, after which serum was separated by centrifugation at 538× *g* for 15 min at 4 °C. Serum levels of TNF-α, IFN-γ, interleukin-1β (IL-1β), and IL-10 were quantified using commercial enzyme-linked immunosorbent assay (ELISA) kits (Shanghai Anxuda Biotechnology Co., Ltd. (Shanghai, China), China; catalog numbers: TNF-α: AXD-M0508, IFN-γ: AXD-M0066, IL-1β: AXD-M0158, IL-10: AXD-M0082). All assays were performed according to the manufacturer’s instructions. Each sample was measured in triplicate, and the mean value was used for analysis.

#### 2.2.4. Western Blotting

Western blot analysis was performed on approximately 50 mg of jejunal tissue. Briefly, tissues were homogenized in RIPA buffer and centrifuged (8611× *g*, 5 min, 4 °C) to collect the supernatant. Proteins (20 μg per lane) were separated by SDS-PAGE alongside a pre-stained marker and transferred to a PVDF membrane. After blocking with 5% BSA for 2 h, the membrane was incubated overnight at 4 °C with primary antibodies against GAPDH (diluted 1:5000), TNF-α (1:500), IFN-γ (1:1000), IL-1β (1:500), and IL-10 (1:1000). Following three 5-min TBST washes, a species-matched HRP-conjugated secondary antibody (1:5000) was applied for 2 h. After the final washes, bands were visualized with an ECL substrate, and images were captured on X-ray film. Band intensity was quantified using Quantity One software (Version 4.6.1; Bio-Rad Laboratories, Hercules, CA, USA), and target protein expression was normalized to GAPDH.

#### 2.2.5. Intestinal Tissue Morphological Observations (H&E Staining and Immunohistochemical Detection)

A jejunal segment located approximately 10–15 cm distal to the ligament of Treitz was collected from each mouse. Tissues were fixed in 4% paraformaldehyde for 24 h, washed in PBS, and trimmed. After sequential dehydration through an ethanol series (75%, 85%, 95%, and 100%), the tissues were cleared twice in xylene (10 min each) and embedded in paraffin. Serial sections of 4-μm thickness were cut using a Leica microtome, mounted on slides, and dried. Following the kit manufacturer’s protocol (Beyotime, Cat#C0105S), sections were stained with H&E, dehydrated, cleared, and mounted with neutral gum. The pathological morphology was then examined and photographed with a microscope (Shanghai Cewei Optoelectronic Technology Co., Ltd., Shanghai, China).

For immunohistochemical staining, paraffin-embedded sections were dehydrated and heated in citrate buffer to retrieve antigens, and endogenous peroxidase activity was blocked with a 3% H_2_O_2_ solution. Then, 5% BSA was used for blocking for 1 h, followed by washing three times with a PBST solution and incubating the sample overnight at 4 °C with primary antibodies against ZO-1, Occludin, Claudin-1, and Mucin-2, respectively. After washing three times with PBST, the samples were incubated with secondary antibodies for 1 h. After removing the secondary antibody solution, DAB chromogenic solution was added for 10 min, followed by washing with water and counterstaining with hematoxylin for 30 s, differentiating for 10 s, and soaking in tap water for 15 min. The sections were dehydrated using graded alcohol and mounted with resin glue, and images were collected under a microscope (Shanghai Cewei Optoelectronic Technology Co., Ltd.). For each section, three fields exhibiting positive DAB staining (yellow or yellowish-brown color) were captured at 200× magnification. The positive area and optical density were calculated using Image-Pro Plus software (Version 6.0; Media Cybernetics, Rockville, MD, USA), and the positive index was subsequently determined (positive index = positive area rate × optical density). The final positive index for each mouse was obtained by averaging the values from the three fields, and the resulting values from individual mice were used for statistical analysis.

#### 2.2.6. SCFA Detection

SCFA analysis was performed by Majorbio Corporation (Shanghai, China). Briefly, small intestinal tissue samples (25 mg) from mice were homogenized in 0.5% phosphoric acid, extracted using 2-ethylbutyric acid as an internal standard, and analyzed on an Agilent 8890B-5977B GC–MS system (Agilent Technologies Inc., Santa, CA, USA) equipped with an HP-FFAP capillary column (30 m × 0.25 mm × 0.25 μm; Agilent J & W Scientific, Folsom, CA, USA). The column temperature program was initiated at 80 °C, increased to 120 °C at rate of 40 °C/min, then to 200 °C at 10 °C/min, and finally held at 230 °C for 3 min. Quantification was performed using selected ion monitoring mode. Calibration curves were performed using eight SCFA standards: acetic, propionic, butyric, isobutyric, valeric, isovaleric, caproic, and isocaproic acids. Detailed analytical procedures and quality control parameters are provided in the Supplementary Materials.

#### 2.2.7. Untargeted Metabolomic Analysis

Untargeted metabolomic analysis was performed by Guangzhou Genedenovo Biotechnology Co., Ltd. (Guangzhou, China). Briefly, tissue samples (50 ± 5 mg) were extracted using methanol and methyl tert-butyl ether. The extracts were separated on a Thermo Vanquish ultra-high-performance liquid chromatography system (Thermo Fisher, Waltham, MA, USA) equipped with an ACQUITY UPLC HSS T3 column (100 mm × 2.1 mm, 1.8 μm; Waters). The mobile phase consisted of aqueous ammonium acetate/acetic acid (A) and acetonitrile (B), delivered at a flow rate of 0.35 mL/min. Mass spectrometric analysis was performed on a Thermo Orbitrap Exploris 120 instrument (Thermo Fisher, USA) in both positive and negative electrospray ionization modes, using full-scan MS (*m*/*z* 70–1050) followed by data-dependent MS/MS acquisition.

Raw data were processed using the XCMS package for peak detection, alignment, and retention-time correction, followed by k-nearest neighbors imputation and probabilistic quotient normalization. Detailed analytical parameters are provided in the Supplementary Materials. Metabolite set overrepresentation analysis was performed using the MSEAp package (version 0.99.0) in R (https://rdrr.io/github/afukushima/MSEAp/ (accessed on 15 March 2025)).

### 2.3. Statistical Analysis

All data were analyzed by one-way analysis of variance (ANOVA) followed by Dunnett’s post hoc test for multiple comparisons. Statistical significance was defined as * *p* < 0.05, ** *p* < 0.01, and *** *p* < 0.001. Sample sizes were not predetermined by a priori power analysis but were instead determined based on empirically validated sample sizes commonly employed in analogous murine models reported in the previous literature.

## 3. Results and Analysis

### 3.1. Safety Assessment of L. murinus in Healthy Mice

**Figure 1:**
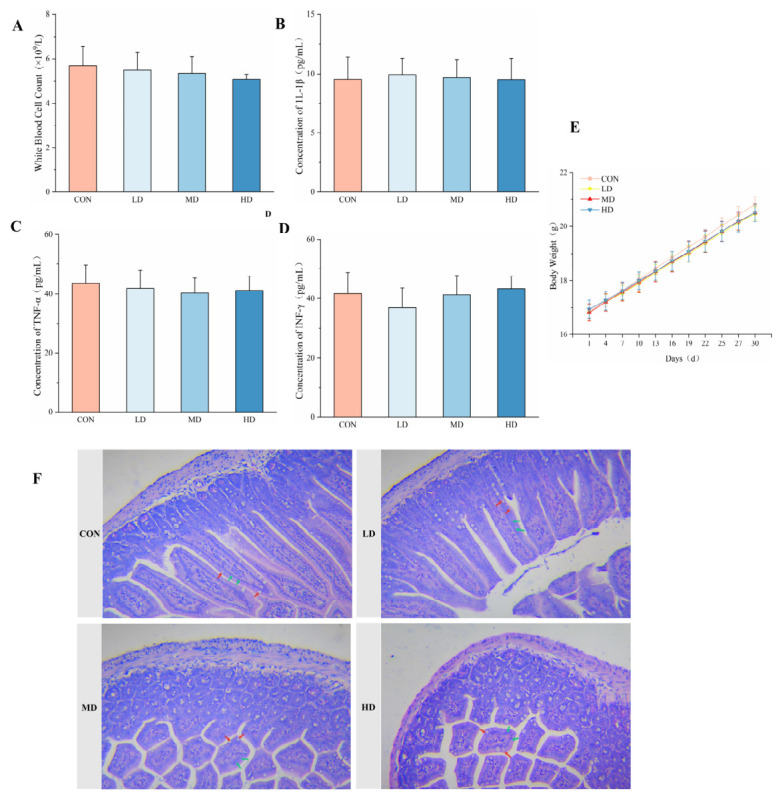
Tolerability assessment of *Lactobacillus murinus* administration in healthy mice. (**A**) Total white blood cell count; (**B**) changes in serum IL-1β concentrations; (**C**) changes in serum TNF-α concentrations; (**D**) changes in serum IFN-γ concentrations; (**E**) changes in body weight; (**F**) H&E staining of intestinal tissue, with red arrows representing goblet cells and green arrows representing absorptive cells. Data are presented as the mean ± SD. All comparisons were performed using one-way ANOVA followed by Dunnett’s post hoc test. No significant differences were observed among the groups (all *p* > 0.05). The groups are represented as follows: CON, saline group; LD, 1 × 10^5^ CFU/mL *L. murinus* treatment group; MD, 1 × 10^7^ CFU/mL *L. murinus* treatment group; HD, 1 × 10^9^ CFU/mL *L. murinus* treatment group.

To evaluate the safety of *L. murinus* in the absence of CTX treatment, a preliminary study was conducted in healthy mice receiving *L. murinus* at low, medium, or high doses, or saline as a control, for 30 days. Based on the whole-blood leukocyte analysis (Figure 1A), no significant abnormalities were detected, indicating that the hematological indicators of the mice were within normal ranges and were not affected by the experimental interventions. Serum cytokine levels (Figure 1B–D) did not differ significantly across groups, suggesting that the experimental treatment did not induce systemic inflammatory responses or other physiological abnormalities. Weight changes for mice in each group are depicted in Figure 1E; no statistically significant differences in body weights were detected between the groups. This suggests that the experimental treatment did not significantly affect mouse growth and development. Additionally, no significant abnormalities were observed in the mice; all exhibited normal activity levels and feeding behaviors, suggesting that *L. murinus* was well tolerated under the conditions tested.

Histologically (Figure 1F), the morphological structures of the small intestine tissues of the examined mice showed no abnormalities, consistent with normal histological characteristics. The villous structure was intact, presenting finger- or tongue-like projections, arranged neatly and densely. The morphology of absorptive cells was regular, appearing columnar, with nuclei located at the base and arranged neatly, and the brush border was clearly visible, indicating that the small intestine had a normal absorptive function. Goblet cells were scattered among them, with clear cytoplasm appearing to be vacuolated and containing mucus and nuclei compressed to the base of the cells; all these features are consistent with normal physiological states for the small intestine. In summary, no significant abnormalities were observed in the body weights, conditions, or physiological indicators among the groups, indicating that the experimental treatment did not adversely influence the growth and physiological state of the mice.

### 3.2. L. murinus Facilitates the Repair of CTX-Induced Damage to the Intestinal Barrier and Regulates the Local Inflammatory Microenvironment

#### 3.2.1. Protective Effects of *L. murinus* on Intestinal Barrier Integrity in CTX-Treated Mice

**Figure 2:**
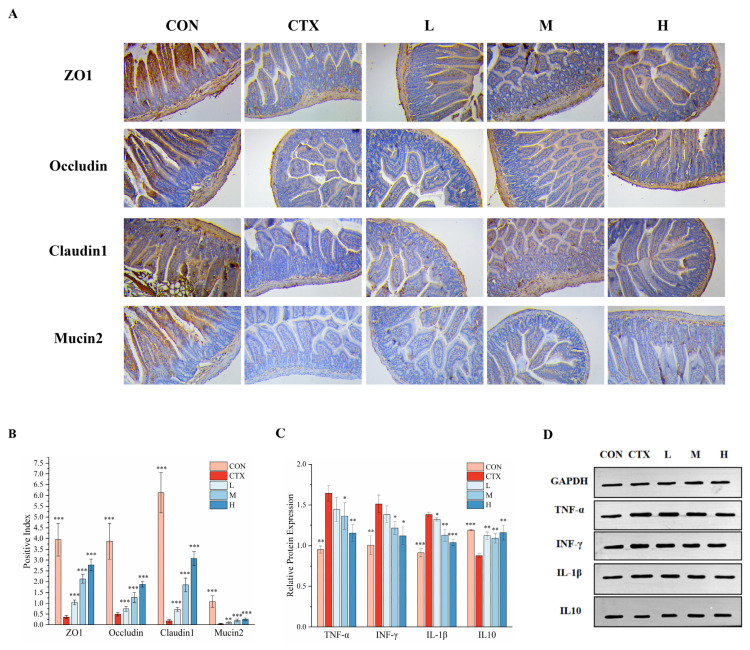
Effects of *Lactobacillus murinus* on the intestinal barrier function and inflammatory factor expression in cyclophosphamide (CTX)-treated mice. (**A**) Immunohistochemical staining of observed changes in ZO-1, Occludin, Claudin-1, and Mucin-2 expression in intestinal tissues; (**B**) semiquantitative analysis of ZO-1, Occludin, Claudin-1, and Mucin-2 protein expression; (**C**) semiquantitative analysis of TNF-α, IFN-γ, IL-1β, and IL-10 inflammatory factor expression; (**D**) representative bands for TNF-α, IFN-γ, IL-1β, and IL-10 protein levels. Data are presented as the mean ± SD. Statistical significance was determined using one-way ANOVA followed by Dunnett’s post hoc test. * *p* < 0.05, ** *p* < 0.01, *** *p* < 0.001 compared with the CTX group. The groups are represented as follows: CON, saline group; CTX, 80 mg/kg CTX treatment group; L, 80 mg/kg CTX + 1 × 10^5^ CFU/mL *L. murinus* treatment group; M, 80 mg/kg CTX + 1 × 10^7^ CFU/mL *L. murinus* treatment group; H, 80 mg/kg CTX + 1 × 10^9^ CFU/mL *L. murinus* treatment group.

To evaluate CTX-induced damage to the physical and chemical barriers of the intestine and the protective effects of *L. murinus*, we detected the expression levels of the tight junction proteins ZO-1, Occludin, and Claudin-1, as well as Mucin-2, a key component of the mucin layer, in the intestinal tissues of mice, through immunohistochemistry (Figure 2A,B). Tight junction proteins are core structures between intestinal epithelial cells that maintain the barrier function [6]. Compared with the control group, CTX administration significantly decreased the expression of all three tight junction proteins. Among them, ZO-1 expression decreased to 9.3% of the control-group levels (0.37 ± 0.08 vs. 3.94 ± 0.75, *p* < 0.001), Occludin expression decreased to 12.7% of these levels (0.49 ± 0.08 vs. 3.87 ± 0.83, *p* < 0.001), and Claudin-1 expression was the most markedly suppressed, decreasing to 3.0% of control-group levels (0.18 ± 0.07 vs. 6.13 ± 0.93, *p* < 0.001), indicating that CTX severely disrupted the tight junction structure of the intestinal epithelium. As the main gel-forming mucin produced by intestinal goblet cells, Mucin-2 forms the gel network of the mucin layer, providing the first chemical barrier against pathogen and toxin invasion [16,17]. Mucin-2 expression in CTX-treated mice was only 3.7% that in the control group (0.04 ± 0.02 vs. 1.08 ± 0.27, *p* < 0.001), suggesting that CTX also severely damaged the chemical barrier function of the intestine.

The *L. murinus* treatment exerted significant and dose-dependent restorative effects on the expression of all four barrier proteins. For tight junction proteins, high-dose *L. murinus* treatment had the greatest restorative effect, restoring ZO-1, Occludin, and Claudin-1 expression to 70.5%, 48.4%, and 50.3% of control levels, respectively (all *p* < 0.001). The medium- and low-dose groups also showed significant trends in restoration, with the degree of recovery positively correlated with the dose. All doses of *L. murinus* significantly increased Mucin-2 expression, with the high-dose group restored to 24.2% of control-group levels (0.26 ± 0.06, *p* < 0.001), which demonstrated superior efficacy compared with the low- and medium-dose groups.

#### 3.2.2. Effects of *L. murinus* on Expression of Intestinal Inflammatory Factors in CTX-Treated Mice

To explore the regulatory effects of *L. murinus* on CTX-induced local intestinal inflammation, we detected the expression levels of key pro-inflammatory mediators (TNF-α, IFN-γ, IL-1β) and the anti-inflammatory cytokine (IL-10) in mouse intestinal tissues via Western blotting (Figure 2C,D). Compared with the normal control group, CTX administration significantly up-regulated the expression of intestinal pro-inflammatory cytokines. Among them, TNF-α levels were increased by approximately 72.6% (1.64 ± 0.09 vs. 0.95 ± 0.04, *p* < 0.001), IFN-γ levels were increased by approximately 50.5% (1.51 ± 0.11 vs. 1.01 ± 0.11, *p* < 0.01), and IL-1β levels were elevated by approximately 51.4% (1.38 ± 0.03 vs. 0.91 ± 0.05, *p* < 0.001). Meanwhile, expression of the anti-inflammatory cytokine IL-10 was significantly suppressed, decreasing by approximately 26.3% (0.88 ± 0.02 vs. 1.19 ± 0.01, *p* < 0.001). Collectively, these findings indicate that CTX treatment induced an intense pro-inflammatory microenvironment in the intestines of mice.

*L. murinus* intervention also reversed this inflammatory imbalance in a dose-dependent manner. For pro-inflammatory factors, the administration of medium and high doses markedly reduced the expression of TNF-α (M: 1.36 ± 0.16, *p* < 0.05; H: 1.15 ± 0.10, *p* < 0.01) and IFN-γ (M: 1.22 ± 0.08, *p* < 0.05; H: 1.12 ± 0.11, *p* < 0.05). Further, IL-1β production was significantly inhibited in all *L. murinus*-treated groups, with the strongest inhibited effect observed in the high-dose group (H: 1.04 ± 0.04, *p* < 0.001), as expression was restored to near-control-group levels. Conversely, the expressions of anti-inflammatory cytokine IL-10 were remarkably elevated across all *L. murinus* treatment groups (L: 1.12 ± 0.05, *p* < 0.01; M: 1.09 ± 0.06, *p* < 0.01; H: 1.16 ± 0.09, *p* < 0.01), with the expression in the high-dose group approaching control-group levels.

#### 3.2.3. Effects of *L. murinus* on SCFA Profiles in CTX-Treated Mice

To investigate the potential link between the immune-regulatory activity of *L. murinus* and changes in gut microbial metabolism, we quantitatively measured levels of seven major SCFA levels in small intestinal contents (Table 1). Levels of acetic acid, as the most abundant SCFA, were significantly decreased in the CTX-treatment group than in normal controls (84.23 ± 7.85 μg/g vs. 162.34 ± 12.46 μg/g, *p* < 0.001). However, *L. murinus* intervention at low, medium, and high doses significantly restored these levels (L group: 76.56 ± 9.41 μg/g, *p* < 0.05; M group: 98.95 ± 9.94 μg/g, *p* < 0.001; H group: 93.66 ± 9.08 μg/g, *p* < 0.05), with the medium-dose group showing the most pronounced recovery effect.

Propionic acid levels were also markedly lower in the CTX-treatment group than in the control group (1.65 ± 0.84 μg/g vs. 4.52 ± 1.06 μg/g, *p* < 0.05). Only high-dose *L. murinus* treatment significantly increased the propionic acid level to 13.77 ± 4.85 μg/g (*p* < 0.01), whereas the low- and medium-dose groups displayed no significant difference compared with the CTX-treatment group. Levels of butyric acid, as a key immune-regulating metabolite, were also significantly suppressed in the CTX treatment group (0.19 ± 0.04 μg/g vs. 2.38 ± 0.69 μg/g, *p* < 0.01). As observed for propionic acid, only high-dose *L. murinus* treatment markedly increased its content to 12.37 ± 2.19 μg/g (*p* < 0.01), far exceeding control levels. Regarding the other analyzed SCFAs, the CTX-treatment group showed downward trends in isovaleric acid, valeric acid, isocaproic acid, and caproic acid levels, compared with the control group. *L. murinus* intervention resulted in different recovery patterns; specifically, valeric acid and caprioc acid levels were significantly restored in all dose groups (*p* < 0.01), whereas isovaleric acid and isocaproic acid levels were significantly elevated only in the medium- and high-dose groups (*p* < 0.05, *p* < 0.001).

### 3.3. L. murinus Reverses CTX-Induced Systemic Immune System Functional Suppression and Imbalances

#### 3.3.1. Effects of *L. murinus* on Splenic Immune Cells in CTX-Treated Mice

**Figure 3:**
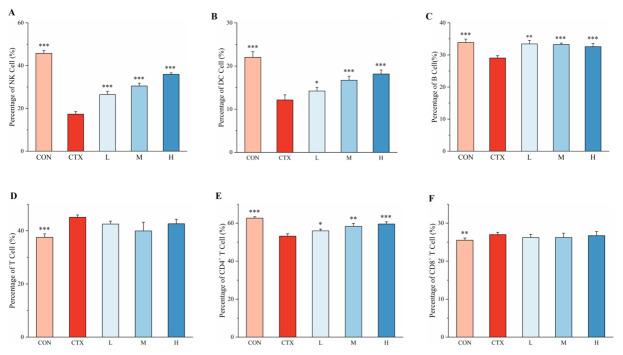
Effects of *Lactobacillus murinus* on splenic immune cells in mice with cyclophosphamide (CTX)-induced immunosuppression. (**A**) NK cell positivity rate; (**B**) dendritic cell (DC) positivity rate; (**C**) B cell positivity rate; (**D**) total T cell positivity rate; (**E**) CD4^+^ cell positivity rate; (**F**) CD8^+^ T cell positivity rate. Data are presented as the mean ± SD. Statistical significance was determined using one-way ANOVA followed by Dunnett’s post hoc test. * *p* < 0.05, ** *p* < 0.01, *** *p* < 0.001 compared with the CTX group. The groups are represented as follows: CON, saline group; CTX, 80 mg/kg CTX treatment group; L, 80 mg/kg CTX + 1 × 10^5^ CFU/mL *L. murinus* treatment group; M, 80 mg/kg CTX + 1 × 10^7^ CFU/mL *L. murinus* treatment group; H, 80 mg/kg CTX + 1 × 10^9^ CFU/mL *L. murinus* treatment group.

To evaluate the regulatory effects of *L. murinus* on innate immune cells, we measured the rates of natural killer (NK) cell and dendritic cell (DC) positivity in mouse spleens by flow cytometry. Compared with that in the control group, the number of NK cells in CTX-treated mice was significantly reduced (17.33 ± 1.18% vs. 45.77 ± 1.30%, *p* < 0.001) (Figure 3A,B). After *L. murinus* intervention, NK cell numbers increased in a dose-dependent manner, with the high-dose group showing the greatest recovery (35.96 ± 0.68%, *p* < 0.001). Similarly, CTX treatment also significantly decreased the number of DC cells (12.15 ± 1.19% vs. 22.02 ± 1.29%, *p* < 0.001). Meanwhile, *L. murinus* treatment reversed this trend, with DC cell positivity rates in the low-, medium-, and high-dose groups significantly higher than those in the CTX group (*p* < 0.05). These results indicate that CTX severely suppressed the function of innate immune cells, but *L. murinus* intervention effectively promoted their recovery.

We further analyzed changes in the proportions of key cell subpopulations associated with acquired immunity. CTX administration significantly reduced the proportion of B cells among lymphocytes (29.04 ± 0.73% vs. 33.89 ± 1.05%, *p* < 0.001) (Figure 3C). However, *L. murinus* treatment effectively restored these proportions across all doses, with the low-dose group approaching control-group levels (33.43 ± 1.11%).

Additionally, CTX treatment induced alteration in the T cell subsets. The total T cell proportion in the CTX group was significantly higher than that in the control (45.07 ± 0.87% vs. 37.52 ± 1.31%, *p* < 0.001) (Figure 3D). Further subgroup analysis showed (Figure 3E,F) that the proportion of CD4^+^ T cells was significantly decreased (53.18 ± 1.25% vs. 62.65 ± 0.73%, *p* < 0.001), whereas the proportion of CD8^+^ T cells was slightly increased (26.98 ± 0.60% vs. 25.51 ± 0.58%, *p* < 0.01), resulting in a decrease in the CD4^+^/CD8^+^ ratio from 2.46 to 1.97. *L. murinus* intervention had clear regulatory effects on this T cell imbalance. The proportion of CD4^+^ T cells showed a dose-dependent recovery, with the proportion in the high-dose group increasing to 59.58 ± 1.13% (*p* < 0.001). However, *L. murinus* did not significantly change the proportion of CD8^+^ T cells (*p* > 0.05). Comprehensive calculations showed that high-dose *L. murinus* treatment restored the CD4^+^/CD8^+^ ratio to 2.23, indicating that this intervention can restore the T cell subgroup imbalance induced by CTX.

#### 3.3.2. Effects of *L. murinus* on Serum Inflammatory Factors in CTX-Treated Mice

**Figure 4:**
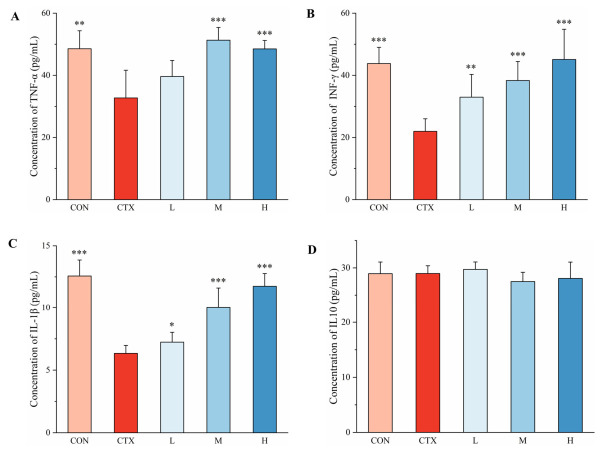
Effect of *Lactobacillus murinus* on serum inflammatory factors in cyclophosphamide (CTX)-treated mice. Serum concentrations of TNF-α (**A**), IFN-γ (**B**), IL-1β (**C**), and IL-10 (**D**) in each group. Data are presented as the mean ± SD. Statistical significance was determined using one-way ANOVA followed by Dunnett’s post hoc test. * *p* < 0.05, ** *p* < 0.01, *** *p* < 0.001 compared with the CTX group. The groups are represented as follows: CON, saline group; CTX, 80 mg/kg CTX treatment group; L, 80 mg/kg CTX + 1 × 10^5^ CFU/mL *L. murinus* treatment group; M, 80 mg/kg CTX + 1 × 10^7^ CFU/mL *L. murinus* treatment group; H, 80 mg/kg CTX + 1 × 10^9^ CFU/mL *L. murinus* treatment group.

To evaluate the systemic inflammatory response triggered by CTX and the effects of *L. murinus*, we performed ELISA to determine the concentrations of key inflammatory cytokines—TNF-α, IFN-γ, and IL-1β—as well as the anti-inflammatory cytokine IL-10 in mouse serum (Figure 4). The CTX-treated mice exhibited a marked reduction in serum levels of the pro-inflammatory cytokines TNF-α, IFN-γ, and IL-1β, compared with the control group. Specifically, TNF-α decreased by 32.6% (32.74 ± 8.90 vs. 48.56 ± 5.78 pg/mL, *p* < 0.01), IFN-γ decreased by 49.8% (21.98 ± 4.04 vs. 43.78 ± 5.24 pg/mL, *p* < 0.001), and IL-1β decreased by 49.6% (6.34 ± 0.64 vs. 12.57 ± 1.29 pg/mL, *p* < 0.001). However, no significant difference in serum levels of the anti-inflammatory cytokine IL-10 was observed between the model and control groups (28.97 ± 1.41 vs. 28.94 ± 2.13 pg/mL, *p* > 0.05).

*L. murinus* intervention reversed the CTX-induced suppression of serum pro-inflammatory factors. Medium and high doses significantly increased TNF-α concentrations to normal levels (M: 51.31 ± 4.09, *p* < 0.001; H: 48.51 ± 2.71, *p* < 0.01), whereas the low-dose group showed an upward trend that was not significant. All doses of *L. murinus* significantly and dose-dependently restored the IFN-γ serum concentration (L: 32.97 ± 7.32, *p* < 0.01; M: 38.33 ± 6.13, *p* < 0.001; H: 45.10 ± 9.72, *p* < 0.001), with the high-dose treatment restoring IFN-γ levels comparable to the control group. Moreover, all doses significantly increased IL-1β levels (L: 7.23 ± 0.79, *p* < 0.05; M: 10.01 ± 1.60, *p* < 0.001; H: 11.74 ± 1.02, *p* < 0.001), showing a dose-dependent effect. Meanwhile, serum IL-10 concentrations remained stable across all experimental groups, with no significant fluctuations.

### 3.4. L. murinus Exerts Protective Effects Through Dose-Dependent Metabolic Regulatory Effects

**Figure 5:**
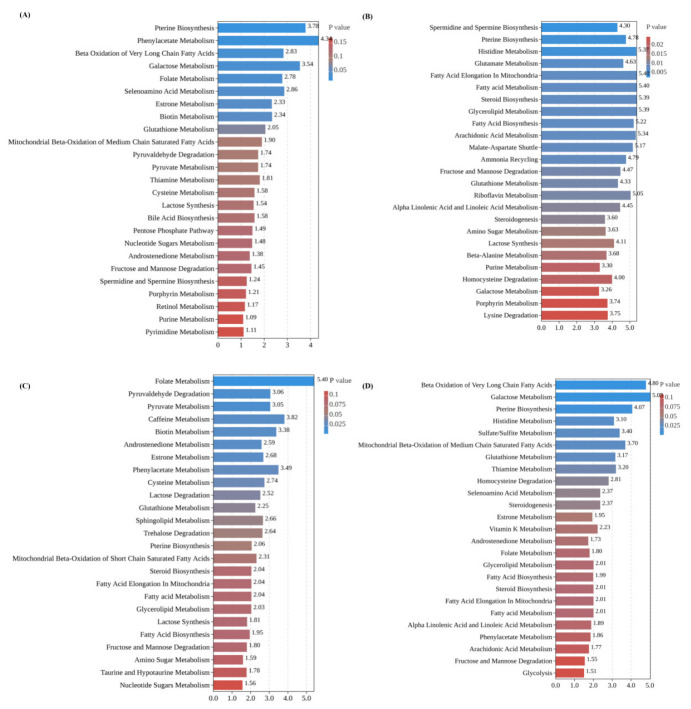
Metabolic regulatory effect of *Lactobacillus murinus* on mice with cyclophosphamide (CTX)-induced immunosuppression. (**A**–**D**) results of metabolic set enrichment analysis (MSEA) based on metabolomics data, with the Y-axis representing the names of significantly enriched metabolic pathways; the X-axis represents the enrichment factor (Enrichment Ratio), indicating the degree of pathway enrichment. (**A**) comparison between the control and CTX groups; (**B**) comparison between the L and CTX groups; (**C**) comparison between the M and CTX groups; (**D**) comparison between the H and CTX groups. Significantly enriched pathways were identified using over-representation analysis with a raw *p*-value threshold of <0.05. The complete MSEA results, including enrichment statistics, raw *p*-values, and lists of contributing metabolites, are provided in the Supplementary Materials. The groups are represented as follows: CON, saline group; CTX, 80 mg/kg CTX treatment group; L, 80 mg/kg CTX + 1 × 10^5^ CFU/mL *L. murinus* treatment group; M, 80 mg/kg CTX + 1 × 10^7^ CFU/mL *L. murinus* treatment group; H, 80 mg/kg CTX + 1 × 10^9^ CFU/mL *L. murinus* treatment group.

Using the Small Molecule Pathway Database (https://smpdb.ca, accessed on 15 March 2025), metabolite set enrichment analysis (MSEA) can help to determine and interpret changes in the concentrations of metabolites associated with important biological pathways [18]. MSEA showed that compared with that in the control group, the serum metabolic profile of CTX-treated mice was systematically disturbed (Figure 5A), with five metabolic pathways significantly enriched (*p* < 0.05). These pathways included the following: pteridine biosynthesis, phenylacetate metabolism, beta-oxidation of very-long-chain fatty acids, galactose metabolism, and folate metabolism. This result suggests that in our model, CTX significantly disturbed the core metabolic networks involved in the one-carbon unit supply, energy metabolism, and aromatic compound transformation.

Low-dose *L. murinus* treatment led to the most extensive changes in metabolic pathways (Figure 5B), with 27 pathways significantly enriched compared with the CTX group (*p* < 0.05). Among them, the spermidine and spermine biosynthesis pathway, which is related to cell proliferation [19,20], was the most significantly altered (*p* < 0.01). Additionally, we identified multiple pathways, including fatty acid metabolism, steroid biosynthesis, glutathione metabolism, and purine metabolism, suggesting that low-dose *L. murinus* may exert systemic protective effects through multi-target mechanisms by regulating antioxidant defenses, lipid homeostasis, and the energy supply.

Medium-dose *L. murinus* treatment affected fewer pathways (eight) but had the most pronounced effect on folate metabolism (*p* < 0.01) (Figure 5C). Additionally, significant changes were observed in androstenedione metabolism, estrone metabolism, and pyruvate metabolism. This suggests that the metabolic effects of the medium-dose intervention may be more focused on specific functional modules related to one-carbon metabolism, endocrine regulation, and glucose metabolism hubs, which may reflect a targeted metabolic repair pattern.

High-dose *L. murinus* treatment resulted in the significant enrichment of seven pathways, with its effects appearing to be primarily associated with energy metabolism-related pathways (Figure 5D). The beta-oxidation of very-long-chain fatty acids (*p* < 0.01) and galactose metabolism (*p* < 0.01) were the two most significantly affected pathways. Additionally, the mitochondrial beta-oxidation of medium-chain saturated fatty acids and sulfate/sulfite metabolism pathways were also significantly altered. This pattern suggests that the metabolic effects of high-dose *L. murinus* may primarily target energy generation and substrate conversion pathways, such as fatty acid oxidation and glucose metabolism.

## 4. Discussion

We systematically explored the protective effects of *L. murinus* on hosts and the underlying mechanisms through the establishment of a CTX-induced mouse model of immunosuppression. Taken together, the results from intestinal histology, immune cell analyses, metabolomics, and cytokine detection demonstrated that CTX treatment simultaneously induced intestinal barrier damage, local inflammation, metabolic dysregulation, and systemic immune imbalance. Notably, *L. murinus* intervention, particularly at medium and high doses, effectively mitigated these pathological features, demonstrating multi-target protective effects and promoting the restoration of host homeostasis.

The integrity of the intestinal barrier is fundamental for maintaining intestinal homeostasis, with core components that include the physical barrier, formed by tight junction proteins (such as ZO-1, Occludin, and Claudin-1), and the chemical barrier, formed by mucin proteins secreted by intestinal goblet cells (mainly Mucin-2). Tight junction proteins are located at the apical side of intestinal epithelial cells, forming a continuous ring-like “belt” structure that maintains the selective permeability of the epithelium by regulating paracellular permeability and preventing the translocation of harmful substances; meanwhile, mucin proteins form a mucus layer covering the intestinal epithelium, through exocytosis, effectively isolating intestinal contents from epithelial cells and providing ecological niches for microbial colonization [21,22]. CTX, as a classic alkylating agent and immunosuppressant, exhibits toxic effects that extend beyond bone marrow suppression, and they are accompanied by severe gastrointestinal reactions [23]. We found that CTX treatment significantly reduces the expression of these key barrier proteins, indicating that it severely disrupts the physical and chemical barrier-associated structures of the intestine, thereby creating a pathological basis for subsequent inflammatory responses and metabolic dysregulation.

Intestinal barrier damage further triggered a strong local inflammatory response, characterized by significant upregulation of the expression of the pro-inflammatory factors TNF-α, IFN-γ, and IL-1β, while that of the anti-inflammatory factor IL-10 was suppressed. This imbalance of intestinal homeostasis also extended to the metabolic level, particularly with the significant dysregulation of SCFA metabolism. SCFAs (such as butyrate, acetate, and pentanoate), as the main end products of dietary fiber fermentation mediated by the gut microbiota, not only provide energy for intestinal epithelial cells but also exert anti-inflammatory and immune-regulatory effects by activating GPCRs (such as GPR43 and GPR109a) or through genetic mechanisms (such as HDAC inhibition) [24]. Among them, butyrate is widely regarded as a key metabolic mediator of the microbiota–host cross-talk, influencing DC phenotypes and T cell differentiation, promoting regulatory T cell and Th1 cell production, and inhibiting excessive Th17 responses [25,26,27]. The CTX-induced decrease in butyrate levels observed in this study may directly diminish protective effects on the intestinal epithelium and systemic immune-regulatory functions, further exacerbating the immune imbalance.

At the systemic level, CTX treatment induced a state characterized by both immune suppression and immune imbalance. The decreased serum levels of pro-inflammatory factors and the reduction in NK cells and DCs in the spleen reflect systemic immune suppression. In contrast, the imbalance in splenic T-cell subpopulations, characterized by a decreased CD4^+^/CD8^+^ ratio, suggests an acquired immune imbalance [28]. The numbers and functions of NK cells, which serve as the first line of defense against infections and tumors, were reduced, potentially impairing immune surveillance capacity [29]. Likewise, the reduction in DCs may compromise the effective initiation of adaptive immune responses [30]. The decrease in serum pro-inflammatory cytokines may result from the direct suppression of immune cell functions by CTX, whereas the translocation of endotoxins or antigens due to intestinal barrier damage may contribute to functional abnormalities and shifts in splenic T-cell subpopulations [31,32]. Furthermore, metabolomic analysis indicated that CTX treatment significantly disrupted key metabolic pathways, including those involved in pteridine metabolism, folate metabolism, and fatty acid β-oxidation, suggesting that metabolic dysregulation may exacerbate impairments in cellular repair and energy homeostasis.

*L. murinus* intervention exerted multi-target and dose-dependent restorative effects on immune homeostasis. In the intestine tract, *L. murinus* dose-dependently enhanced the expression of tight junction proteins and mucins, repairing the intestinal barrier structure. Simultaneously, it effectively inhibited the release of pro-inflammatory cytokine, and promoted production of the anti-inflammatory IL-10, thereby alleviating mucosal inflammation. Notably, *L. murinus* significantly increased SCFA levels in the intestinal contents, with the high-dose treatment having the most pronounced restorative effects on butyrate and propionate levels. The high-dose-specific surge in butyrate levels likely stems from a threshold-dependent, lactate-mediated cross-feeding network. As a homolactic fermenter, high-dose *L. murinus* may provide sufficient lactate to serve as a preferred substrate for endogenous butyrate-producing commensals, such as, *Eubacterium*, *Roseburia*, and *Anaerobutyricum*, thereby promoting butyrate production [33,34]. This interpretation is consistent with recent evidence demonstrating that lactate availability directly stimulates butyrate synthesis by gut microbiota through cross-feeding interactions [34]. The results of this study support the core role of butyrate in mediating the immune-regulatory effects of probiotics; specifically, high-dose *L. murinus* increased butyrate levels and most effectively promoted the expression of IL-10 in the intestine and restored the proportion of CD4^+^ T cells and the CD4^+^/CD8^+^ ratio in the spleen, suggesting a “metabolism–immune axis” regulatory pathway, from metabolic regulation to the immune balance.

At the systemic immune level, *L. murinus* treatment resulted in characteristics of “restoring homeostasis” rather than simply enhancing immunity. Further, it reversed the state of systemic immune suppression induced by CTX, restoring serum pro-inflammatory factors to near physiological levels, while effectively increasing the proportions of NK cells, DCs, B cells, and CD4^+^ T cells and readjusting the total T cell and CD8^+^ T cell proportions, ultimately promoting normalization of the imbalanced CD4^+^/CD8^+^ ratio. This multi-target and balance-enhancing regulatory pattern reflects the ideal characteristics of *L. murinus* as an immune regulator.

Metabolomic analysis further revealed the dose-dependent metabolic regulatory effects of *L. murinus* treatment, with each dose exhibiting synergistic protective effects through different metabolic pathways. The low-dose intervention primarily affected glutathione metabolism and purine metabolism pathways. The identification of glutathione, as the most important intracellular antioxidant, suggests that *L. murinus* may enhance cellular oxidative stress-defense capabilities, alleviating CTX-induced reactive oxygen damage [35]. The regulatory effect on purine metabolism may influence the nucleotide pool balance and DNA repair processes, which are particularly critical for rapidly proliferating intestinal epithelial cells and immune cells [36]. The predominant effects of the medium-dose intervention were associated with the restoration of folate metabolism and steroid hormone metabolism. Folate, as a key cofactor involved in one-carbon metabolism, supports normal DNA synthesis and methylation modifications, potentially facilitating cell proliferation and differentiation [37,38]. The regulatory effect on steroid hormone metabolism may affect the regulation of systemic inflammatory responses through modulation of the synthesis of anti-inflammatory hormones, such as glucocorticoids, echoing the observed anti-inflammatory effects [39,40]. The effects of the high-dose intervention were clearly directed toward energy metabolism, particularly promoting the β-oxidation of very-long-chain fatty acids and galactose metabolism. Enhanced fatty acid β-oxidation may provide a sufficient ATP supply for high-energy-utilizing cells and tissues, such as immune cells, and may generate acetyl-CoA that can enter the tricarboxylic acid cycle to support cellular biosynthetic needs [41,42]. Galactose metabolism is an important pathway for energy supply and may play a key role in maintaining overall metabolic balance by influencing glycogen synthesis and glycosylation processes [43]—consistent with the significant recovery of systemic immune function observed in the high-dose group. Notably, this dose-dependent metabolic regulatory pattern was progressive, from basic defense (low dose) to specialized repair (medium dose) to the energy supply (high dose). The effects of each *L. murinus* dose, through different but complementary metabolic pathways, collectively formed a multi-level protective network against CTX metabolic toxicity, suggesting a possible explanation for the observed dose-dependent immune-protective effects. These associations suggest that dose-specific metabolic pathways may contribute to immune restoration; however, the current untargeted metabolomics analysis does not provide direct evidence of causality. Further experimental studies are required to validate these findings and establish causal relationships.

A critical limitation of this study is the insufficient verification of the mechanism associated with the key metabolite, butyrate. Although we observed that *L. murinus* intervention could significantly restore intestinal butyrate levels and that changes in butyrate were highly correlated with protective effects, such as intestinal barrier repair, the upregulation of IL-10 expression, and the restoration of the CD4^+^/CD8^+^ ratio in the spleen, this study did not provide direct evidence of butyrate mediating these protective effects. Further, we did not determine whether exogenous butyrate supplementation could mimic the protective effects of *L. murinus*, and we did not use SCFA receptor antagonists or gene knockout animal models to verify that known butyrate receptor pathways, such as GPR43/GPR109a, are necessary for this effect. Therefore, whether butyrate plays a core mediating role in this process or whether this is merely a concomitant phenomenon still requires further clarification. Elucidating this mechanism is crucial to understanding how probiotics regulate host immunity through distinct metabolites.

This study has several limitations that should be acknowledged.

First, we did not directly assess immune cell infiltration in intestinal tissues. Although CTX treatment significantly upregulated the expression of pro-inflammatory cytokines (TNF-α, IFN-γ, and IL-1β) in the intestine, indirectly indicating local inflammation, we did not perform immunostaining for specific immune cell markers (e.g., CD3 for T cells, F4/80 for macrophages, and MPO for neutrophils). Therefore, whether *L. murinus* directly inhibits the recruitment and accumulation of immune cells in the intestinal mucosa remains to be determined.

Second, we did not conduct time-course experiments to identify the peak of CTX-induced intestinal inflammation or systemic immunosuppression. Consequently, we cannot definitively determine whether the inflammatory and immunosuppressive phenotypes observed on day 30 represent the peak, plateau, or early recovery phase. Nevertheless, the 30-day endpoint remains appropriate for evaluating the restorative effects of *L. murinus*, as the CTX group continued to exhibit significant local inflammation (elevated TNF-α, IL-1β, and IFN-γ levels) and systemic immunosuppression (reduced splenic NK cells, DCs, CD4^+^ T cells, and serum cytokines levels) compared with the control group. Future studies incorporating systematic time-course analyses and direct immune cell staining are warranted to further elucidate the dynamic progression of CTX-induced injury and the local immunomodulatory mechanisms of *L. murinus*.

Third, the MSEA used in this study identifies pathways enriched with altered metabolites but does not provide information on pathway directionality (i.e., whether a pathway is globally upregulated or downregulated). Moreover, within key pathways such as glutathione, folate, and fatty acid β-oxidation, individual metabolites exhibited inconsistent directional changes across treatment doses, making simple pathway-level interpretations potentially misleading. Therefore, the MSEA findings should be considered hypothesis-generating. Targeted metabolomics and metabolic flux analyses are needed to accurately characterize the directional regulation of these pathways.

Fourth, several additional issues warrant consideration. This study focused on the acute recovery phase following chemotherapy; thus, the long-term biological effects of sustained supraphysiological SCFA levels induced by *L. murinus* remain unclear. In addition, the proposed lactate-mediated cross-feeding mechanism is primarily inferred from metabolite profiles and microbiota composition data. Direct validation using metagenomic, metatranscriptomic, or functional approaches is required to confirm the underlying metabolic interactions. Furthermore, all findings were derived from a murine model, and the clinical applicability of *L. murinus* intervention must be verified in human studies. Despite these limitations, our findings provide robust preclinical evidence that targeting the metabolism–immune axis through *L. murinus* administration may represent a promising strategy for promoting immune reconstitution following chemotherapy.

## 5. Conclusions

*L. murinus* alleviates CTX-induced immune damage through multidimensional synergistic mechanisms. Locally in the intestine, *L. murinus* effectively restores barrier protein levels, inhibits pro-inflammatory factor production, enhances expression of the anti-inflammatory factor IL-10, and restores SCFA levels, demonstrating clear local anti-inflammatory effects. At the systemic level, it restores the proportions of NK cells, DCs, and CD4^+^ T cells, adjusts CD8^+^ T cell levels, and improves the immune cell balance, while regulating multiple metabolic pathways, such as those associated with glutathione, folate, and fatty acid oxidation, providing support for immune recovery and achieving a systemic regulatory effect. This dual strategy of local anti-inflammatory and systemic immune-regulatory effects not only alleviates chemotherapy-related intestinal mucositis but also helps to improve systemic immune suppression, demonstrating its potential clinical value as an adjunctive intervention for chemotherapy.

## Figures and Tables

**Table 1:** Comparison of small intestine short-chain fatty acid levels in mice of each group (μg/g).

Group	Acetic Acid	Propionic Acid	Butyric Acid	Isovaleric Acid	Valeric Acid	Isocaproic Acid	Caproic Acid
CON	162.34 ± 12.46 ***	4.52 ± 1.06 *	2.33 ± 0.69 **	0.39 ± 0.07	0.31 ± 0.10 *	10.97 ± 2.13 *	0.64 ± 0.13 *
CTX	34.23 ± 7.85	1.65 ± 0.84	0.19 ± 0.04	0.28 ± 0.06	0.07 ± 0.01	4.32 ± 1.41	0.33 ± 0.02
L	76.56 ± 9.41 **	3.60 ± 1.28	0.15 ± 0.09	0.38 ± 0.09	0.15 ± 0.02 **	8.51 ± 2.97	0.52 ± 0.05 **
M	98.95 ± 9.94 ***	6.68 ± 0.90 *	0.44 ± 0.10 *	0.46 ± 0.04 *	0.27 ± 0.06 **	12.42 ± 0.60 ***	0.45 ± 0.05 *
H	93.66 ± 9.08 *	13.77 ± 4.85 **	12.37 ± 2.19 **	0.88 ± 0.36 *	0.33 ± 0.02 ***	15.42 ± 1.05 ***	0.65 ± 0.12 **

Note: Data are presented as the mean ± SD (*n* = 7 per group). Statistical significance was determined using one-way ANOVA followed by Dunnett’s post hoc test. * *p* < 0.05, ** *p* < 0.01, *** *p* < 0.001 compared with the CTX group. Abbreviations: CON, control; CTX, cyclophosphamide; L, low-dose *Lactobacillus murinus* treatment; M, medium-dose *L. murinus* treatment; H, high-dose *L. murinus* treatment.

## Data Availability

The original contributions presented in this study are included in the article/Supplementary Materials. Further inquiries can be directed to the corresponding author.

## Supplementary Materials

The following supporting information can be downloaded at: https://www.mdpi.com/article/10.3390/biom16070957/s1, Figure S1: Original western blot images; Note S1: Method of SCFA detection and untargeted metabolomic analysis; Table S1: Detailed results of MSEA.

## Abbreviations

The following abbreviations are used in this manuscript:

CTX	Cyclophosphamide
HDACs	histone deacetylases
GPCRs	G protein-coupled receptors
TNF-α	tumor necrosis factor-alpha
IFN-γ	interferon-gamma
IL-10	interleukin-10
ECL	enhanced chemiluminescence
H&E	hematoxylin–eosin
DCs	Dendritic cells
NK	Natural killer
ANOVA	analysis of variance
MSEA	metabolite set enrichment analysis
