# Supplementary material for: Lactobacillus murinus Mediates Multi-Target Protection to Alleviate Cyclophosphamide-Induced Intestinal Injury and Immune Suppression Through the Gut–Metabolism–Immune Axis
Source: Biomolecules. 2026 Jun 29;16(7):957. doi: 10.3390/biom16070957 (PMC13407138; doi:10.3390/biom16070957)
Supplement: Supplementary file 1 [file biomolecules-16-00957-s001.zip › Note S1 Method of SCFA detection and untargeted metabolomic analysis.pdf]

Lactobacillus murinus **mediates multi-target protection to alleviate cyclophosphamide-induced intestinal injury and immune suppression through the gut–metabolism–immune axis**

Supplementary Material

1. SCFA detection

1.1 Preparation of SCFA standard solution

High-performance liquid chromatography-grade n-butanol was used as a solvent to prepare mixed standard stock solutions and internal standard stock solutions. The mixed standard stock solution contained eight SCFAs, specifically acetic acid, propionic acid, butyric acid, isobutyric acid, pentanoic acid, isopentanoic acid, caproic acid, and isohexanoic acid, at concentrations of 6000, 3000, 500, 500, 500, 1000, 500, and 500 μg/mL, respectively. The internal standard stock solution was an n-butanol solution of 2-ethylbutyric acid at a concentration of 1000 μg/mL.

Using n-butanol as a diluent, the aforementioned two stock solutions were mixed and diluted stepwise to prepare a series of standard working solutions containing nine concentration points to establish a calibration curve. The concentration of the internal standard 2-ethylbutyric acid was kept constant at 10 μg/mL in all working solutions. The specific gradients of each SCFA at the nine concentration points were as follows: acetic acid (0.018, 0.48, 4.8, 24, 144, 300, 350, 490, and 600 μg/mL); propionic acid (0.024, 0.24, 2.4, 12, 72, 130, 180, 240, and 300 μg/mL); butyric acid, isobutyric acid, pentanoic acid, hexanoic acid, and isohexanoic acid (all 0.004, 0.04, 0.4, 2, 12, 25, 30, 40, and 50 μg/mL); isopentanoic acid (0.008, 0.06, 0.8, 4, 24, 50, 60, 80, and 100 μg/mL). All solutions were stored at −20°C in the dark and equilibrated to room temperature and thoroughly mixed before analysis.

1.2 Sample processing

Mouse small intestine contents was collected, and 25 mg was weighed into a 2 mL grinding tube, with 500 μL of water (containing 0.5% phosphoric acid) added, and the sample was cryogenically ground for 3 min (50 Hz) twice, followed by ultrasonic treatment for 10 min and centrifugation at 8611 × *g* at 4°C for 15 min. The entire supernatant was transferred to a 1.5 mL centrifuge tube, and 0.2 mL of the internal standard stock solution was added for extraction. The mixture was vortexed for 10 s, subjected to low-temperature ultrasound for 10 min, and centrifuged at 13000 × *g* at 4°C, for 5 min. The supernatant was then transferred to a sample vial for analysis.

1.3 Gas chromatography-mass spectrometry detection

The analysis was performed using an 8890B-5977B GC/MSD gas chromatography–mass spectrometry system (Agilent Technologies Inc. CA, USA). Chromatographic conditions were as follows: HP FFAP capillary column (30 m × 0.25 mm × 0.25 μm, Agilent J&W Scientific, Folsom,

CA, USA); carrier gas, high-purity helium (purity not less than 99.999%); flow rate, 1.0 mL/min; injection port temperature, 260°C. The injection volume was 1 μL, with split injection at a split ratio of 10:1 and solvent delay of 2.5 min. The temperature program was as follows: the initial temperature of the column oven was 80°C, ramping to 120°C at 40°C/min, then to 200°C at 10°C/min, and holding at 230°C for 3 min. The electron impact ion source was set at an ion source temperature of 230°C, quadrupole temperature of 150°C, and transfer line temperature of 230°C, with electron energy at 70 eV. Scanning was performed in selected ion monitoring mode.

1.4 Data analysis

Target SCFA ion fragments were automatically identified and integrated using Masshunter quantification software (Agilent Technologies, version: v10.0.707.0), with manual checks for assistance. The concentrations in each sample were calculated using the internal standard method based on the standard curve, converting values to the actual SCFA contents in the samples.

2 Untargeted metabolomic analysis

2.1 Sample preprocessing

Approximately 50 mg of the tissue samples (±5 mg) was accurately weighed and added to 200 μL of a pre-chilled 50% methanol aqueous solution and thoroughly homogenized using a homogenizer. Then, 600 μL of pre-chilled methyl tert-butyl ether solution was added, and the sample was vortexed and centrifuged at 3000 rpm at 4°C for 15 min. Next, 200 μL of the upper organic phase was transferred to a new centrifuge tube and freeze-dried under a vacuum. After drying, the residue was re-dissolved in 200 μL of a dichloromethane–methanol solution (1:1, v/v) and centrifuged again at 538 × *g* for 15 min, and the supernatant was taken for analysis. During the experiment, an equal amount of supernatant from each sample was mixed to prepare a quality control sample.

2.2 Chromatography–mass spectrometry conditions

For the chromatography system, a Thermo Vanquish ultra-high-performance liquid chromatography system (Thermo Fisher, USA) was used for separation with an ACQUITY UPLC HSS T3 column (100 mm × 2.1 mm, 1.8 μm; Waters). The column temperature was set to 40°C; mobile phase A was an aqueous solution containing 5 mmol/L ammonium acetate and 5 mmol/L acetic acid, and mobile phase B was acetonitrile. The flow rate was 0.35 mL/min, and the injection volume was 4 μL. The gradient elution program was as follows: 0–0.8 min, 2% B; 0.8–2.8 min, 2% to 70% B; 2.8–5.0 min, 70% to 90% B; 5.0–5.5 min, 90% to 100% B; 5.5–7.5 min, maintained at 100% B; 7.5–7.6 min, 100% to 2% B; 7.6–10.0 min, maintained at 2% B.

For the mass spectrometry system, a Thermo Orbitrap Exploris 120 high-resolution mass spectrometer (Thermo Fisher, USA) equipped with an electrospray ion source was used. The ion source temperature was set at 350°C; the sheath gas flow rate was 50 Arb, the auxiliary gas flow

rate was 15 Arb, and the sweep gas flow rate was 1 Arb. Data from positive and negative ion modes were collected separately, with capillary voltages set at +3.8 kV and −3.4 kV, respectively. Full scan-data-dependent acquisition mode was used, as follows: the first-level full scan range was m/z 70–1050, with a resolution of 60,000; within each scanning cycle, the top four precursor ions with the highest response intensity (intensity threshold >5000) were selected for second-level fragmentation scanning, with a second-level resolution of 15,000. The dynamic exclusion time was set to 4 s.

2.3 Data processing and analysis

Raw data were converted to mzML format using ProteoWizard software. Peak extraction, alignment, and retention time correction were performed using the XCMS program, with key parameters set as follows: for peak extraction, the centWave algorithm (m/z deviation = 20 ppm, peak width range = 5–25 s, signal-to-noise ratio threshold = 6) was used; peak grouping parameters (bw = 5, binSize = 0.015) were used. For the extracted peak area data, quality control was first performed as follows: metabolite feature peaks with a missing rate >50% in quality control samples or >80% in experimental samples were removed. Subsequently, missing values in retained feature peaks were filled using the KNN method, and outliers were deleted; finally, data were normalized using PQN (Probabilistic Quotient Normalization) to ensure comparability between samples and metabolites. Bioinformatic analysis was performed using Omicsmart, a dynamic real-time interactive online platform for data analysis (https://www.omicsmart.com).
