# Supplementary figures and images for: Lactobacillus murinus Mediates Multi-Target Protection to Alleviate Cyclophosphamide-Induced Intestinal Injury and Immune Suppression Through the Gut–Metabolism–Immune Axis
Source: Biomolecules. 2026 Jun 29;16(7):957. doi: 10.3390/biom16070957 (PMC13407138; doi:10.3390/biom16070957)

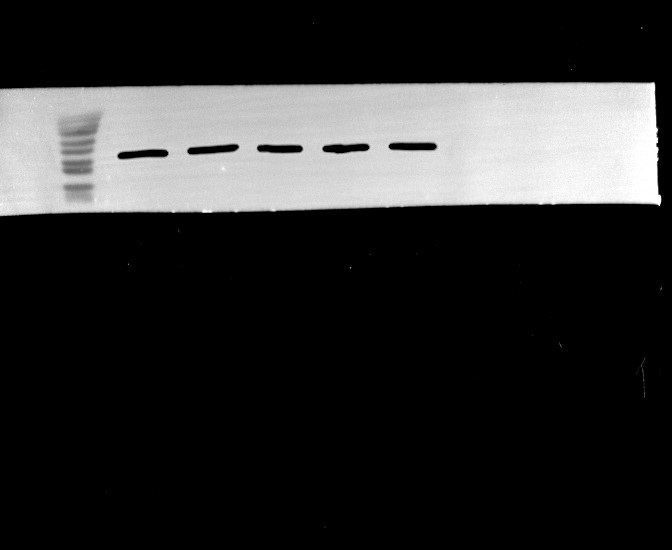

Supplement: Supplementary file 1 [file biomolecules-16-00957-s001.zip › Figure S1_ Original western blot images/gapdh-1.jpg]

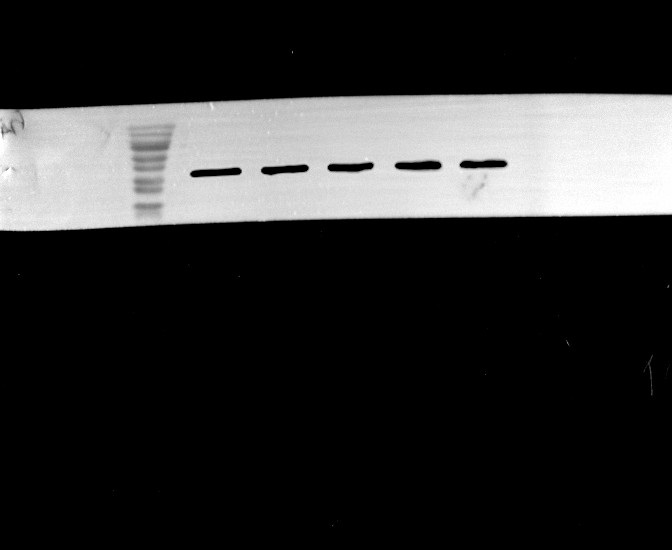

Supplement: Supplementary file 1 [file biomolecules-16-00957-s001.zip › Figure S1_ Original western blot images/gapdh-2.jpg]

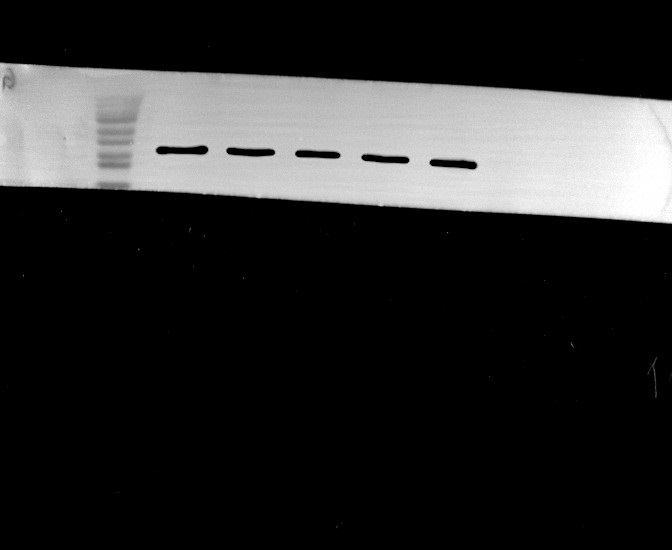

Supplement: Supplementary file 1 [file biomolecules-16-00957-s001.zip › Figure S1_ Original western blot images/gapdh-3.jpg]

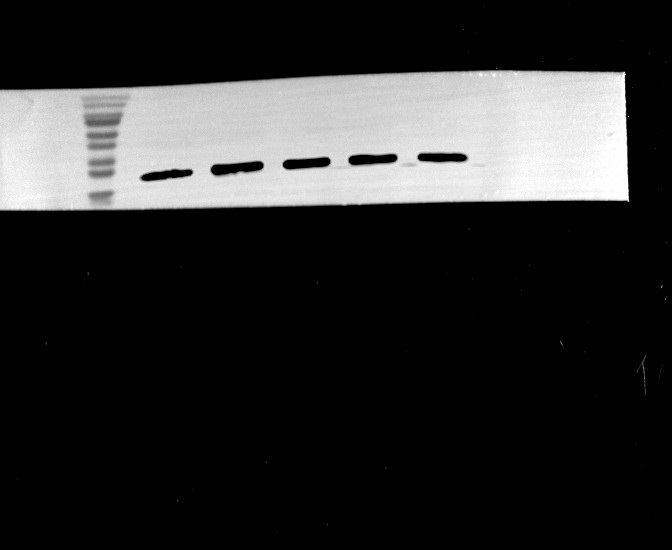

Supplement: Supplementary file 1 [file biomolecules-16-00957-s001.zip › Figure S1_ Original western blot images/ifnr-1.jpg]

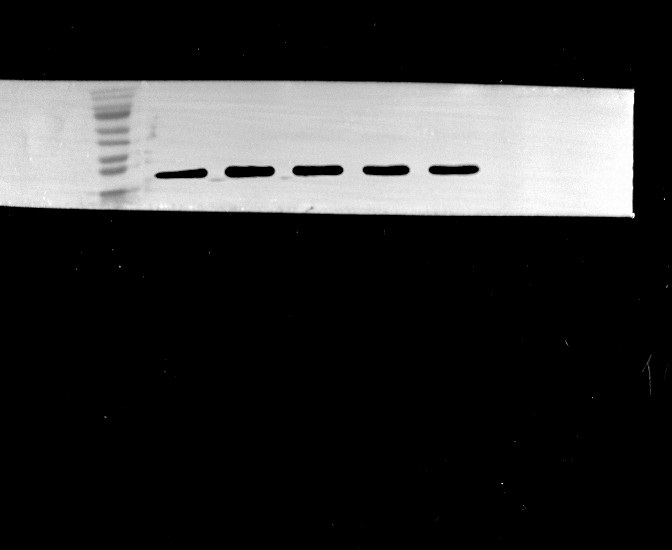

Supplement: Supplementary file 1 [file biomolecules-16-00957-s001.zip › Figure S1_ Original western blot images/ifnr-2.jpg]

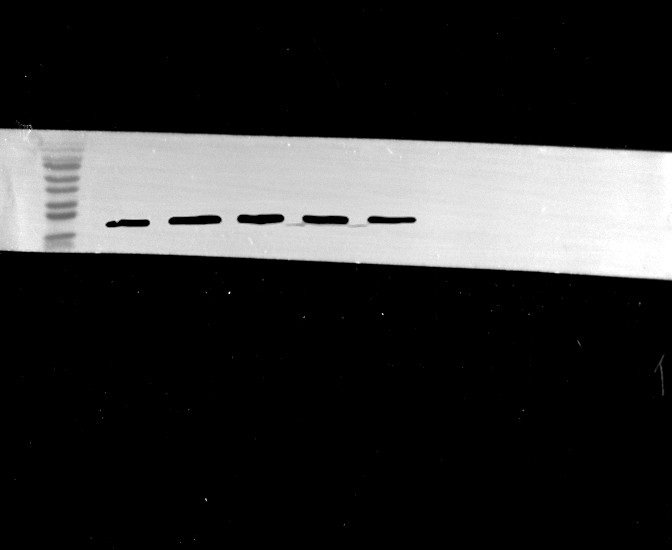

Supplement: Supplementary file 1 [file biomolecules-16-00957-s001.zip › Figure S1_ Original western blot images/ifnr-3.jpg]

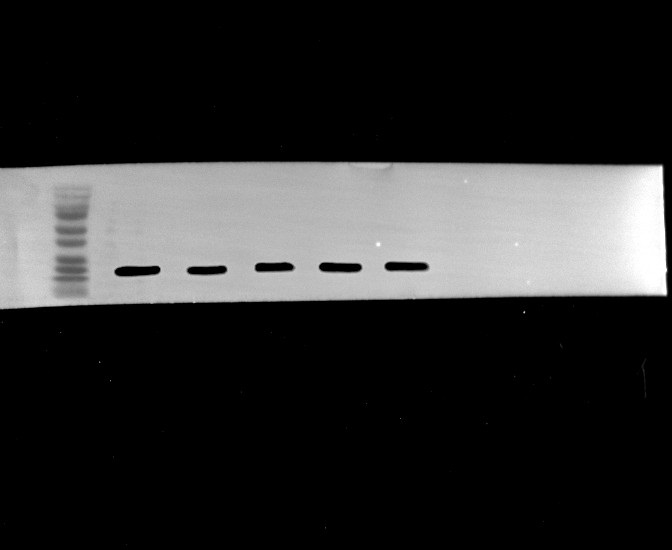

Supplement: Supplementary file 1 [file biomolecules-16-00957-s001.zip › Figure S1_ Original western blot images/il10-1.jpg]

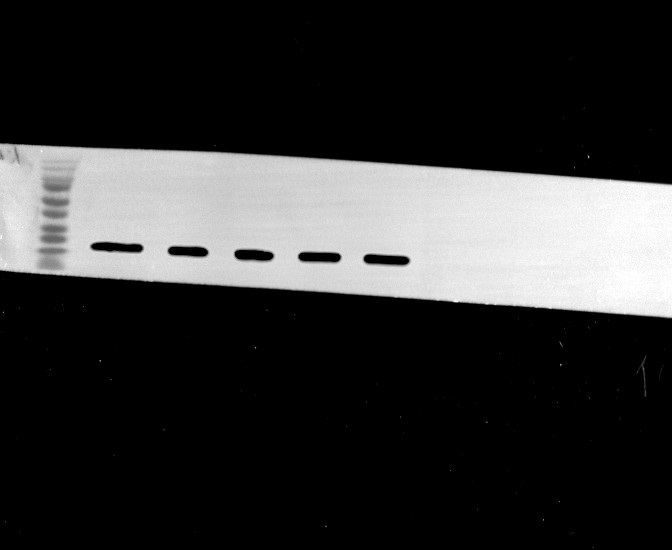

Supplement: Supplementary file 1 [file biomolecules-16-00957-s001.zip › Figure S1_ Original western blot images/il10-2.jpg]

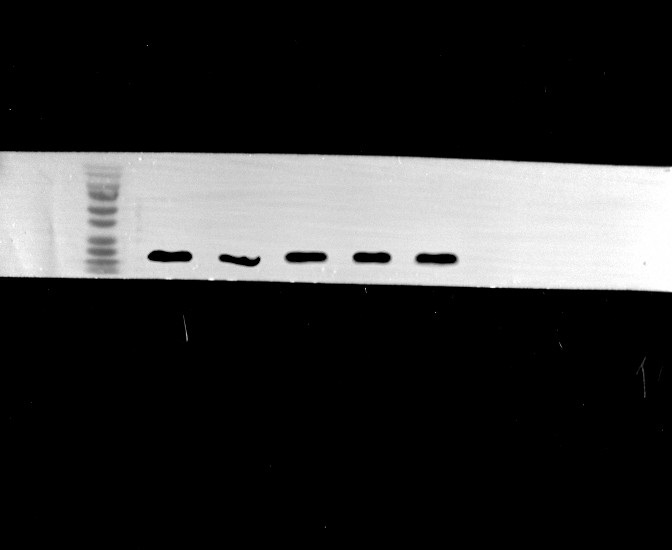

Supplement: Supplementary file 1 [file biomolecules-16-00957-s001.zip › Figure S1_ Original western blot images/il10-3.jpg]

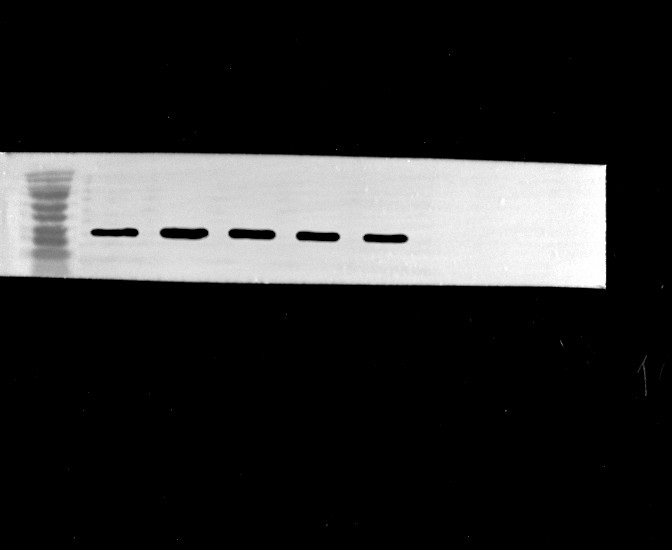

Supplement: Supplementary file 1 [file biomolecules-16-00957-s001.zip › Figure S1_ Original western blot images/il1b-1.jpg]

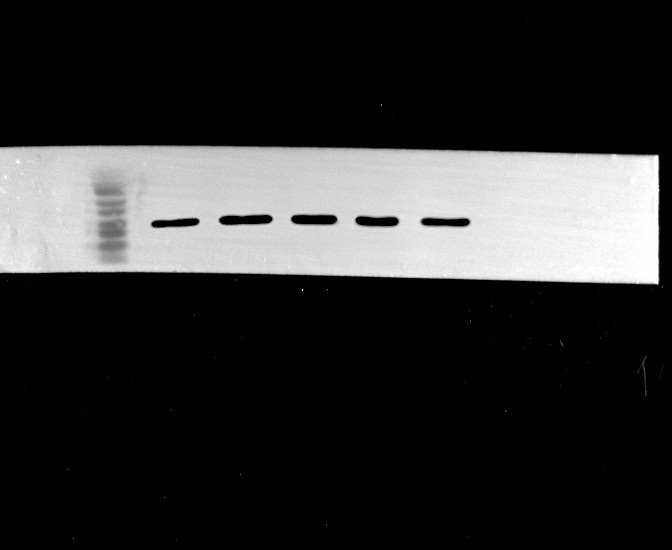

Supplement: Supplementary file 1 [file biomolecules-16-00957-s001.zip › Figure S1_ Original western blot images/il1b-2.jpg]

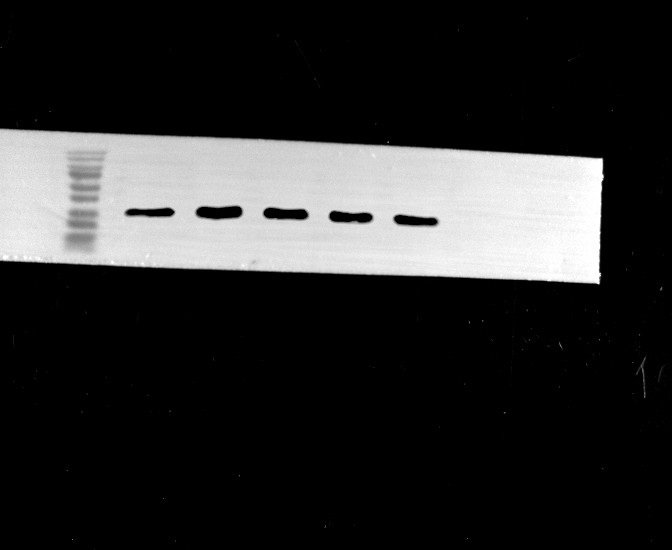

Supplement: Supplementary file 1 [file biomolecules-16-00957-s001.zip › Figure S1_ Original western blot images/il1b-3.jpg]

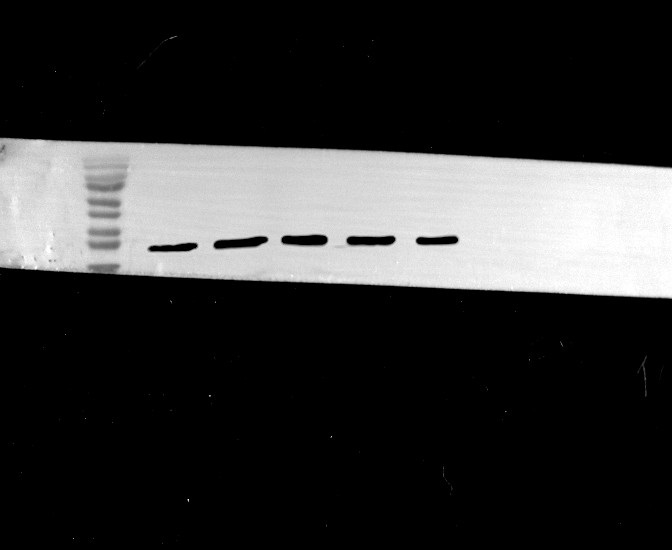

Supplement: Supplementary file 1 [file biomolecules-16-00957-s001.zip › Figure S1_ Original western blot images/tnfa-1.jpg]

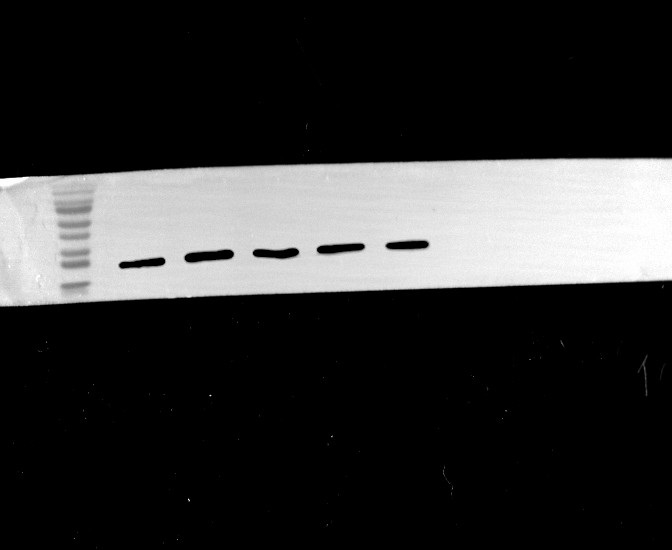

Supplement: Supplementary file 1 [file biomolecules-16-00957-s001.zip › Figure S1_ Original western blot images/tnfa-2.jpg]

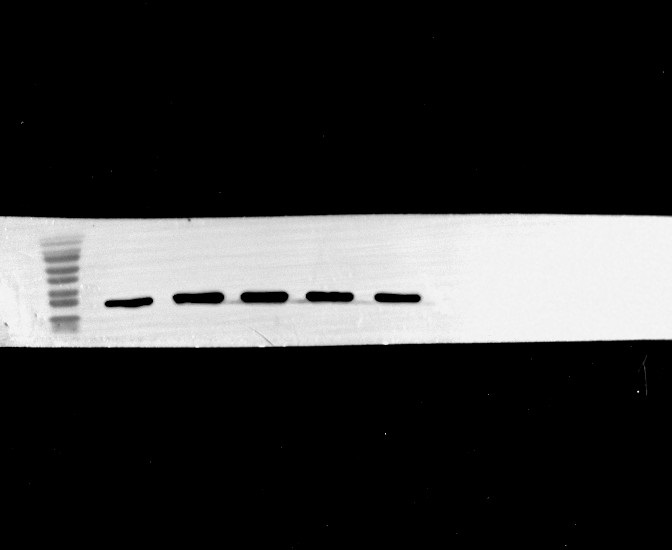

Supplement: Supplementary file 1 [file biomolecules-16-00957-s001.zip › Figure S1_ Original western blot images/tnfa-3.jpg]
